# Towards the Continuous Hydrothermal Synthesis of ZnO@Mg_2_Al-CO_3_ Core-Shell Composite Nanomaterials

**DOI:** 10.3390/nano10102052

**Published:** 2020-10-16

**Authors:** Ian Clark, Jacob Smith, Rachel L. Gomes, Edward Lester

**Affiliations:** 1 Advanced Materials Research Group, Faculty of Engineering, University of Nottingham, University Park, Nottingham NG7 2RD, UK; ian.clark@nottingham.ac.uk (I.C.); jacob.smith@nottingham.ac.uk (J.S.); 2 Food, Water, Waste Research Group, Faculty of Engineering, University of Nottingham, University Park, Nottingham NG7 2RD, UK; Rachel.gomes@nottingham.ac.uk

**Keywords:** continuous hydrothermal synthesis, layered double hydroxide, composite, nano hybrid structures, Ultraviolet (UV) properties

## Abstract

Core-shell Zinc Oxide/Layered Double Hydroxide (ZnO@LDH) composite nanomaterials have been produced by a one-step continuous hydrothermal synthesis process, in an attempt to further enhance the application potential of layered double hydroxide (LDH) nanomaterials. The synthesis involves two hydrothermal reactors in series with the first producing a ZnO core and the second producing the Mg_2_Al-CO_3_ shell. Crystal domain length of single phase ZnO and composite ZnO was 25 nm and 42 nm, respectively. The ZnO@LDH composite had a specific surface area of 76 m^2^ g^−1^, which was larger than ZnO or Mg_2_Al-CO_3_ when produced separately (53 m^2^ g^−1^ and 58 m^2^ g^−1^, respectively). The increased specific surface area is attributed to the structural arrangement of the Mg_2_Al-CO_3_ in the composite. Platelets are envisaged to nucleate on the core and grow outwards, thus reducing the face–face stacking that occurs in conventional Mg_2_Al-CO_3_ synthesis. The Mg/Al ratio in the single phase LDH was close to the theoretical ratio of 2, but the Mg/Al ratio in the composite was 1.27 due to the formation of Zn_2_Al-CO_3_ LDH from residual Zn^2+^ ions. NaOH concentration was also found to influence Mg/Al ratio, with lower NaOH resulting in a lower Mg/Al ratio. NaOH concentration also affected morphology and specific surface area, with reduced NaOH content in the second reaction stage causing a dramatic increase in specific surface area (> 250 m^2^ g^−1^). The formation of a core-shell composite material was achieved through continuous synthesis; however, the final product was not entirely ZnO@Mg_2_Al-CO_3_. The product contained a mixture of ZnO, Mg_2_Al-CO_3_, Zn_2_Al-CO_3_, and the composite material. Whilst further optimisation is required in order to remove other crystalline impurities from the synthesis, this research acts as a stepping stone towards the formation of composite materials via a one-step continuous synthesis.

## 1. Introduction

Layered double hydroxides (LDHs) are a type of anionic clay. LDHs conform to a general formula: $\left[M_{1-x}^{\mathrm{II}}M_{x}^{\mathrm{III}}\left({\mathrm{OH}}_{2}\right)\right]{\left(A^{n-}\right)}_{x/n}{.\mathrm{zH}}_{2}O$ [1], where M^II^ and M^III^ represent bivalent (Mg^2+^, Ca^2+^, Co^2+^, Zn^2+^, Cu^2+^, Ni^2+^) and trivalent metal ions (Al^3+^, Co^3+^, Fe^3+^, Cr^3+^, Ni^3+^ Co^3+^) are distributed in brucite-like sheets [2]. A^n−^ is representative of interlayer anions, which hold together the positively charged brucite-like sheets through electrostatic force. A figure detailing this structure can be found elsewhere [3]. Layered double hydroxides have been used as adsorbents for pollutant mitigation [4,5,6,7], drug delivery [8] flame-retarding polymers [9,10], catalysts [11,12], and as precursor materials for catalysts [13,14]. 

LDH core-shell materials could offer an interesting extension of current uses, since the LDH shell and core materials are joined without the need for binding agents. The core-shell composites could be tuned to have specific functionalities, such as photocatalysis combined with selective adsorption or drug release combined with magnetic properties. Magnetic core-shell LDHs have been used as drug delivery vehicles, produced via a two-step synthesis process whereby the magnetic ferrite core was produced in the first step, and then, the LDH shell was grown via a co-precipitation reaction step [15,16]. More recently, however, a variety of core nanoparticles have been incorporated into composites following the same two-stage synthesis protocol. Silica spheres surrounded by MgAl-CO_3_ shells have been used to remove the anti-inflammatory drug diclofenac from wastewater [17,18]. Zeolites have also been employed as core materials for adsorbent composites for water treatment as a way to combine the high specific surface area of zeolites with the ion exchange functionality of LDHs [19,20]. It has been found that the micro-pore volume of the zeolite was reduced when the shell was synthesised in a zeolite@LDH composite [19]. To date, two stage hydrothermal synthesis reactions have been used primarily to produce nanowires on substrates [21,22,23,24,25]. Co_3_O_4_ composites have been applied in electronics and the two-step batch hydrothermal method is preferred, as it provides materials as readymade electrodes [21,22]. ZnO@LDHs have also been utilised in photocatalytic applications along with TiO_2_@LDHs. TiO_2_@CoAl-CO_3_ and ZnO@CuAl-CO_3_ core-shell materials have been shown to exhibit water splitting capabilities for H_2_ production [25,26,27,28]. Likewise, ZnO/MgAl-CO_3_ composites have been used for the photodegradation of dyes such as Malachite Green and Methylene Orange [29,30]. By altering the LDH in composites from MgAl-CO_3_ to ZnCr-CO_3_ type, magnetic core-shell composites have also been applied to adsorption and photocatalysis of methylene blue [31]. To produce core-shell materials, reactions have been typically carried out in multi-step processes, either by hydrothermal or co-precipitation reactions. Two-step synthesis methods for core-shell composite materials can be time consuming and to the knowledge of the authors, there have been no attempts to incorporate both reactions into a single process. 

The aim of this research was to produce ZnO@Mg_2_Al-CO_3_ core-shell nanomaterials via a once through continuous hydrothermal synthesis method, negating the need for multiple step processes. This continuous hydrothermal method has been utilised for the controlled synthesis of both metal oxides and LDHs (Dunne et al., 2015; Clark et al., 2018) individually and was used as a framework to design a system where rapid synthesis of both the ZnO core and LDH shell could be achieved in a single step.

## 2. Materials and Methods 

### 2.1. Reagents and Chemicals

All chemicals for precursor solutions were reagent grade and were used as received. Aluminium nitrate (Al(NO_3_)_3_·9H_2_O ≥ 99%) and were purchased from Acros Organics (Geel, Belgium). Sodium carbonate (Na_2_CO_3_ ≥ 99%), zinc nitrate (Zn(NO_3_)_2_. 2H_2_O ≥ 98%), magnesium nitrate (Mg(NO_3_)_2_·6H_2_O ≥ 98%), and sodium hydroxide (NaOH ≥ 98%) were purchased from Sigma Aldrich (Dorset, United Kingdom). Prior to synthesis, fresh solutions were prepared by dissolving metal salts in deionised water.

### 2.2. Preparation of Composite Nanomaterials

**Figure 1:**
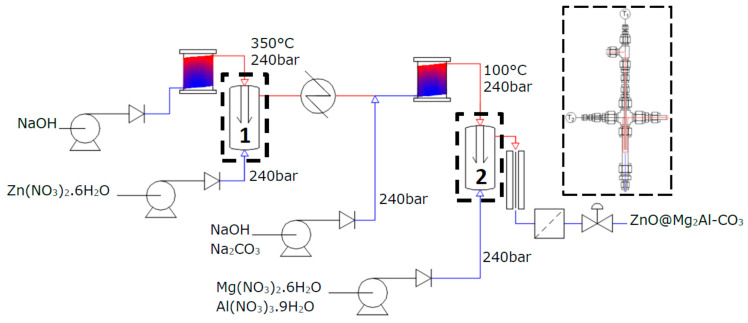
Simplified reaction schematic for sequential counter current flow system with reactor 1 and reactor 2 labelled 1 and 2, respectively.

Attempted continuous hydrothermal synthesis (CWS) of composite nanomaterials was carried out in a sequential reaction system that combines two counter current flow reactors (University of Nottingham, Nottingham UK). The counter current reactor design is detailed elsewhere [32]. The first reactor is used to produce the ZnO core, and the second is used to produce the LDH shell. For synthesis Zn(NO_3_)_2_.6H_2_O (0.05 M) was fed at ambient temperature, in the up-flow to the first reactor at 10 mL min^−1^, while a solution of NaOH (0.05 M) was heated to 350 ± 5 °C and fed in the down-flow at 20 mL min^−1^. The outflow was then cooled in a heat exchanger before NaOH (0.15 M) and Na_2_CO_3_ (0.017 M) was fed into the flow, via a third pump again at 10 mL min^−1^. This mixed feed was heated to 100 ± 3 °C in the down-flow into the second reactor. LDH metal salts were pumped into the up-flow for the second reactor, at ambient temperature, at 20 mL min^−1^. The reaction is outlined in a simplified schematic (Figure 1). Reaction pressure for the whole system was maintained at 240 bar by a Back Pressure Regulator (Pressure Tech, Hadfield, UK). Samples were centrifuged and washed thoroughly. Following washing samples were freeze-dried. To compare the composite ZnO@Mg_2_Al-CO_3_ (labelled ZnO-LDH) with the pure materials, separate ZnO and Mg_2_Al-CO_3_ materials were also synthesised. 

Reactors 1 and 2 consisted of a 1/8” tube inside a 3/8” tube and a 1/4” tube inside a 1/2”, respectively. The increase in the inner tube diameter in reactor 2 was necessary to prevent blockages at the nucleation point at the outlet of the inner tube as a result of solid particles flowing in from reactor 1. A series of other experiments were carried out to investigate the influence of flow rates in the two reactors and the potential for unreacted precursors from the first reactor influencing the products forming in the second reactor. The variation in reactor geometry and flow rates between reactor 1 and reactor 2 system result in changes to the Reynolds number and mixing regime between the first and second reactors. Any variation in LDH synthesis between reactor 1 and reactor 2, as a result of increased flow rate and changes to reactor geometry, were examined through changes in material characteristics. Mg_2_Al-CO_3_ was produced at 60 mL min^−1^ with deionised water flowing from reactor 1 and LDH precursors flowing into reactor 2. The precursor concentrations were altered to accommodate the change in flow rate and so the ratio of OH^−^ to M^II^ + M^III^ was kept at 2.5. The effect of residual counter ions from the synthesis of ZnO (Unreacted NO_3_^−^ and Zn^2+^) were also investigated using the dual reactor setup with synthesis of Mg_2_Al-CO_3_ only occurring in the second reactor. Table 1 and Figure S1 in Supplementary information outline the details of the full matrix of synthesis experiments.

### 2.3. Materials Characterisation

X-ray diffraction (XRD) of powder samples was carried out on a Bruker D8-Advance Diffractometer (Bruker, Billerica, MA, USA)using Cu Kα radiation (λ = 0.15418 nm) between 5° and 70° 2θ, at a rate of 0.75° min^−1^. Crystal domain lengths were calculated using the Scherrer equation. Scanning Electron Microscopy with Energy Dispersive Spectroscopy (SEM-EDS) was used to establish composition of ZnO-LDH; prior to analysis, samples were pelleted and carbon-coated to produce a flat surface. Analysis was performed using a Siemens XL30 SEM (Siemens, Munich, Germany)with a tungsten filament. Specific surface area and pore size distribution were analysed using nitrogen adsorption at 77 K with a Micromeritics Tristar II 3020 (Micromeritics, Atlanta, GA, USA)The Brunauer–Emmett–Teller (BET) and Barrett–Joyner–Halenda (BJH) methods were used to calculate surface and pore size distribution. Samples were dispersed onto copper supported lacy carbon films (Agar Scientific) and a JEOL 2000FX (JEOL UK, Welwyn Garden City, UK)transmission electron microscope (TEM) was used to image particles with acceleration voltage at 200 kV. Image analysis of 100–200 individual particles from these TEM micrographs were then used to calculate average particle size and particle size distribution. These data were compared against the crystal domain length (CDL) calculated from the Scherrer equation. Photo-activity of materials was examined using a Cary 300 Ultraviolet-Visible (UV-Vis) spectrophotometer (Agilent, Stockport, UK),scanning between 800 nm and 200 nm. Thermal stability was assessed on a TA Q500 TGA instrument (TA Instruments, Elstree, UK). Analysis heating rate was 5 °C min^−1^ between 25 °C and 700 °C under N_2_ flow at 40 mL min^−1^. The conversion rate of Zn^2+^ to ZnO in reactor 1 experiments was measured via atomic absorption spectroscopy (AAS) (after removing ZnO particles from the product slurry using a centrifuge) using a Perkin Elmer 272 Atomic Adsorption Spectrophotometer with a Zn hollow cathode lamp (λ = 213.9).

## 3. Results and Discussion

### 3.1. Crystal Characterisation of Composite and Single-Phase Materials

**Figure 2:**
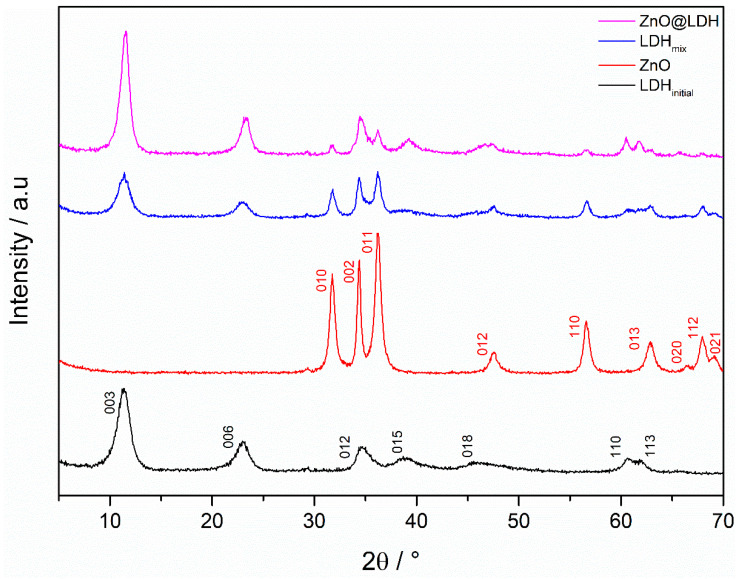
X-ray diffractograms for ZnO@LDH, ZnO and Mg_2_Al-CO_3_ produced in reactor 1 (LDH_initial_) and a mix of LDH_initial_ and ZnO (LDH_mix_).

Pure phase Mg_2_Al-CO_3_ exhibits R$\bar{3}$m rhombohedral crystal symmetry, whereas ZnO exhibits P6_3_mc symmetry. The diffraction pattern for ZnO-LDH shows reflections for both ZnO and Mg_2_Al-CO_3_ phases (Figure 2), indicating either formation of ZnO-LDH or a mix of both ZnO and Mg_2_Al-CO_3_ phases. The relative intensity of LDH reflections in the ZnO-LDH pattern suggests a more crystalline hydrotalcite phase in the proposed composite material when compared with LDH_initial_, this is not the case with the ZnO, as there are no changes in relative reflection intensity between the ZnO and ZnO-LDH diffractogram. The lattice parameters (Table 2) show slight variation between pure materials and the composites. However, the discrepancy is minimal, and the lattice parameters generally correspond well with values in literature [33,34,35,36,37].

Crystal domain length (CDL) approximated from the Scherrer equation (Table 3) indicates differences between single phase profiles and those in the composite. The average crystallite size of the ZnO phase in the composite material is significantly larger than in single phase ZnO (42 nm from 25nm, respectively). Crystal size of the LDH phase in the composite is marginally larger than that of LDH_initial_. The larger ZnO CDL in ZnO-LDH may be due to the fact that some Zn^2+^ remains unconverted following the first reaction. Elemental analysis of reaction liquor indicates that conversion of Zn^2+^ to ZnO is approximately 94 ± 1%. The remaining Zn^2+^ may then enable existing ZnO particles to grow further. 

**Figure 3:**
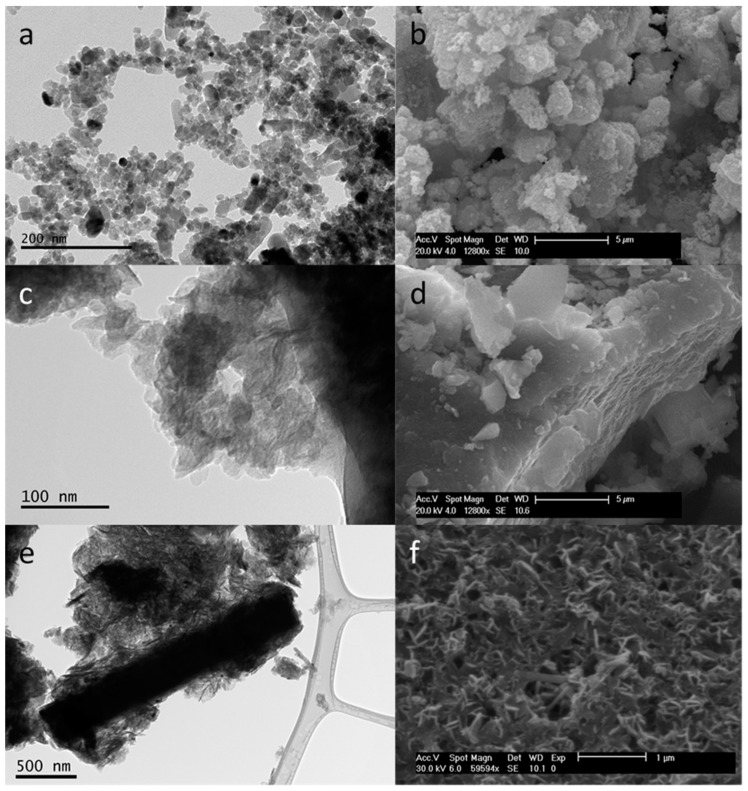
Transmission electron microscopy images (200 kV and ×150,000–500,000 magnification) (**a**) ZnO, (**c**) LDH_initial_, (**e**) ZnO-LDH and SEM micrographs (**b**) ZnO, (**d**) LDH_initial_, (**f**) ZnO-LDH.

Electron microscope images in Figure 3 outline the differences between the three materials under scrutiny. The LDH platelets shown in Figure 3c are less defined in terms of shape, compared with the ZnO nanoparticles with a significant degree of agglomeration and stacking (Figure 3d). The Mg_2_Al-CO_3_ platelets in the ZnO-LDH sample appear to exhibit less face stacking than the platelets in the LDH_initial_ sample. There are also rods visible in the SEM micrograph in Figure 3f. There are larger LDH platelets evident from TEM in the ZnO-LDH sample compared with LDH_initial_, and the CDL for (110) reflection is also slightly larger. The increase in size of crystals may be due to a growth-dominated reaction. Platelets first nucleated using ZnO as seed points then grow preferentially rather than nucleate to form new platelets. Having said this, the differences in CDL for LDH platelets between LDH_initial_ and ZnO-LDH could equally be as a result of the changes in flow rates of synthesis, affecting the Reynolds numbers and mixing regime. ZnO crystals are significantly larger in both XRD and TEM in the composite materials compared to the pure ZnO due to continued growth from unreacted Zn^2+^ as well as Ostwald ripening inside the second reactor. The TEM micrograph in Figure 3e shows a large rod particle surrounded by Mg_2_Al-CO_3_ platelets. The micrograph might indicate that the formation of a composite material has been achieved, as a result of areas of high pH within the reaction system. The higher pH (> 10) led to a negative zeta potential on the ZnO allowing an electrostatic attraction to form between the ZnO and Mg_2_Al-CO_3_ [17,38].

### 3.2. Specific Surface Area Analysis

**Figure 4:**
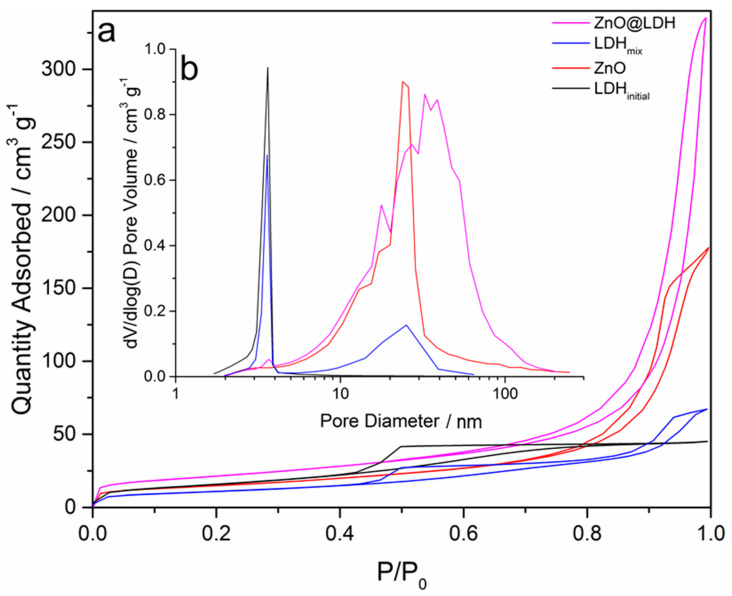
(**a**) Type-IV isotherms, (**b**) pore size distribution for ZnO@LDH composite, ZnO, Mg_2_Al-CO_3_ (LDH_initial_), and a dry mix of ZnO and LDH_initial_ (LDH_mix_).

Type-IV shape isotherms can be identified in Figure 4 for all materials. The ZnO isotherm demonstrates a H1 type hysteresis loop, indicating meso-porosity with ink bottle pore shapes, which is most likely to arise from the agglomeration of the ZnO (Figure 3a). From the S_BET_ analysis of the isotherms (Table 4), the ZnO-LDH appears to have a higher specific surface area than either pure phase material (ZnO and LDH_initial_) or the artificial mix of the two pure materials (LDH_mix_). TEM and SEM images (Figure 3) show some 3D structuring in the ZnO-LDH sample (Figure 3f), which is less evident in LDH_initial_ (Figure 3d). The LDH_initial_ samples appears to exhibit large block-like particles with distinct flat sheets. The more open 3D structure of the platelets in the ZnO-LDH (resulting from edge-face connections), gives rise to a H1 hysteresis loop indicating that there are some aggregated LDH platelets present in the sample [39]. The LDH_initial_ shows more face–face stacking in material, which might explain the H2 and H3 hysteresis profile in the LDH_initial_ isotherm. The sharp desorption branch is associated with H2 hysteresis and the inclined tail at high P/P_0_ found in H3 loops. This is to be expected as H3 hysteresis is associated with aggregation of platelet-like particles and clays [39]. It is also worth noting that there is a significant difference between the ZnO-LDH and LDH_mix_ (the mix of separate samples of LDH and ZnO) isotherm types. The isotherm for LDH_mix_ shows a similar profile to those given by the ZnO and LDH_initial_ samples. These differences in profile, specific surface areas from BET, and pore volume (Table 4) suggest that ZnO-LDH is a genuine composite material rather than a binary mix of discrete ZnO and Mg_2_Al-CO_3_ particles.

### 3.3. Chemical Characterisation 

The molecular ratio of magnesium to aluminium was calculated using SEM-EDS analysis (Figure S2a–d, Table S1 and was found to be 1.97 for LDH_initial_. This value is close to the Mg^2+^/Al^3+^ ratio in the precursor solutions. However, the Mg/Al ratio in the composite material was found to be 1.27 using SEM-EDS analysis. This change indicates a lower conversion of Mg^2+^ in the composite compared to the LDH. This can be seen in Figure S2c,d; the intensity of the EDX peaks in Figure S2c indicate approximately a 2:1 ratio of counts for magnesium to aluminium, however the same peaks in Figure S2d show that the levels of Mg and Al were similar. The reduced NaOH in the synthesis of ZnO-LDH flowing into the second reactor could cause the reduced Mg^2+^/Al^3+^ ratio. Unreacted Zn^2+^ may also react in the presence of OH^−^ and Al^3+^ to produce Zn_x_Al-CO_3_, which is the most likely cause of the reduced Mg^2+^ conversion. The unreacted Zn^2+^ (in the precursor) is approximately 6%, determined by atomic absorption spectroscopy, on the reaction liquor from ZnO synthesis. This low amount of unreacted Zn^2+^ may contribute to the formation of Zn_2_Al-CO_3_ LDH that would unlikely be detectable during XRD analysis. It is also possible that ZnO particles in the reactor down-flow may partially re-dissolve, as a result of increased temperature and reduced pH of the solution when the LDH metal salts are added to the second reactor [40]. This would allow Zn^2+^ in the reactor to form Zn_x_Al-CO_3_. Increasing the NaOH content in reactor 2 by increasing the concentration in feed stream 3, which is after the first reactor but before the second heater (Figure 1), could resolve this issue, as the addition of more NaOH would not increase the conversion of Zn^2+^ to ZnO, but it would reduce any dissolution of the ZnO. However, it is possible that it would lead to greater precipitation of Mg^2+^ [41] and nucleation and growth rates of the LDH in the second reactor.

### 3.4. Thermal Stability

With LDH decomposition, there are three distinct mass loss events, dependant on LDH type. These mass loss events correspond to the peaks denoted in a dW/dT plot:

(a) The loss of surface bound moisture (<100 °C) [42].

(b) The bound interlayer water (100–200 °C) [43].

(c) The breakdown of the layered structure and loss of interlayer anion (in this case -CO_3_ to the oxide and CO_2_) [44].

**Figure 5:**
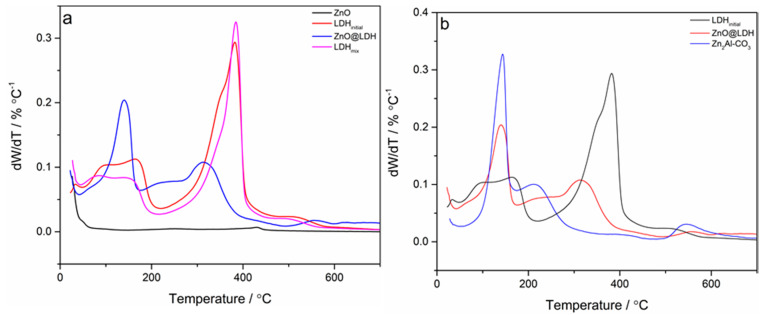
Derivative thermal decomposition of (**a**) ZnO, LDH_initial_, LDH_mix_, and ZnO-LDH (**b**) Zn_2_Al-CO_3_ ZnO-LDH and LDH_initial_. All samples heated to 700 °C with a rate of 5 °C min^−1^ 40 mL min^−1^ flow rate of N_2_.

These samples were dried prior to analysis, which means that the first peak is not particularly obvious. The TGA mass loss profile for the ZnO-LDH sample is significantly different from the pure ZnO, LDH_initial_ and LDH_mix_ samples (Figure 5a). There is a pronounced peak around 150 °C that is evident in the ZnO-LDH profile but not the others. In principle, this peak should be visible in one of the, ZnO or Mg_2_Al-CO_3_, constituent materials components. However, the peak is evident in the mass loss profile for Zn_2_AlCO_3_ LDH. Zn_2_AlCO_3_ has a less well defined third peak around 220 °C, which is smaller and at a lower temperature than the peaks at 375 °C seen in the LDH_initial_ and 320 °C with the ZnO-LDH sample (Figure 5b). A Zn_2_Al-CO_3_ LDH was produced as part of other synthesis work relating to continuous LDH synthesis [40].

The breakdown of the lamella structure and consequent mass loss of interlayer anions in ZnO-LDH suggests the thermal stability is of the layered double hydroxide within the ZnO-LDH composite is somewhat less than the LDH. This is possibly due to the presence of Zn_x_Al-CO_3_ and a compromised Mg_2_Al-CO3 LDH structure, arising from a lower Mg/Al ratio. This might also be caused (to some extent) by the smaller particle size of the LDH component in the ZnO-LDH sample. The total mass loss of the ZnO-LDH during heating was 37%, whereas the mass loss of the LDH was 47% and ZnO was 3%. The mass loss of the composite material indicates that the mass fraction of the LDH in the composite is approximately 0.77. This value is based on theoretical mass loss based on combining single phase ZnO and pure LDH1 at different ratios. The mass fraction of LDH derived from the TGA profile was larger than the mass fraction given by the SEM-EDS analysis. This may be in part due to the presence of Zn_x_Al-CO_3_ in the composite material.

### 3.5. Electronic Structure Characterisation

Direct transitions were found for all three materials tested using the Tauc equation and the UV profiles (Figure S3 in ESI). The composite material shows a slight increase in band gap energy compared with the ZnO (Table 5), meaning that a lower wavelength of light, and more energy, is necessary for electron transition to occur. LDH_initial_ has a band gap energy of 5.24 indicating poor semiconducting characteristics. The other three are relatively similar.

### 3.6. The Impact of Reactor 1 vs. Reactor 2

Several experiments were undertaken to understand the influence of secondary production of the LDH material and the presence of the ZnO in the inlet flow to reactor 2. It is worth noting that the LDH_initial_ itself was manufactured in reactor one using the standard flow conditions 20 mL min^−1^ down flow and 10 mL min^−1^ up flow. As such, the LDH_mix_ and ZnO are also products from reactor 1. As such, these experiments were specifically to “deconstruct” the processes at work in the cascaded reactor set up where ZnO is manufactured in the first reactor and LDH is manufactured in the second. 

### 3.7. LDH Formation in Reactor 1 and in Reactor 2

The main difference between the two reactors is the mixing dynamics, which is more turbulent in reactor 2 (albeit still laminar), compared to reactor 1 (see Table 6). This suggests that immediately after the initial mixing of the down-flow and up-flow streams, mixing is reduced to a greater extent in reactor 1 than in reactor 2. The changes in flow regime appear to have a small impact on the diffraction pattern of LDH_initial_ and LDH3 (Figure S4 in ESI), with LDH_initial_ showing more defined reflections. This is unsurprising considering the small difference in Reynolds numbers. The CDL of the samples appears to be impacted slightly by the change in Reynolds number as the CDL in the (110) plane is increased by 5nm from LDH_initial_ to LDH3 (Table 6). 

The more turbulent flow regime in reactor 2 appears to have a significant negative effect on specific surface area (S_BET_ is reduced to <5 m^2^ g^−1^ for LDH3). The cause of this is unclear, however it may be due to previously unreacted OH^−^ causing a greater degree of agglomeration and stacking in the sample due to the increased mixing occurring across the length of the reactor. It is unlikely that the marginal increase in CDL has caused the dramatic decrease in S_BET_ and pore volume (Table 7). LDH3 exhibits a more defined “block-sheet” type structuring with no visible faults or porous structures (Figure S5 in ESI), but this does not directly explain the drop in specific surface area.

### 3.8. The Impact of Residual Ions from ZnO Synthesis

As discussed earlier, the conversion of Zn^2+^ to ZnO in reactor 1 was approximately 94%. This equates to 72 ± 6mg L^−1^ of unreacted Zn^2+^. There is also the NO_3_^−^ counter ion from the Zn precursor in the reaction liquor that is used to produce the ZnO-LDH composite. Diffractograms show decreased crystallinity for samples produced with lower NaOH concentration (Figure S6). The lower crystallinity is caused by disruption in the layered structure by the reduced Mg(OH)_2_ (Table 8). This would suggest that, as a higher crystallinity is observed for ZnO@LDH compared to LDH_initial_ (Figure 2), a lower NaOH concentration in reactor 2 is unlikely to be the cause of the lower Mg^2+^/Al^3+^ ratio observed in SEM-EDS analysis. The CDL outlined in Table 9 indicates only a marginal increase with the samples produced at increased NaOH conditions. Residual Zn^2+^ ions appear to have little effect on the crystal structure because the concentration in the feed is so low. Nitrate also appears to have no effect on the structure of the samples. The effect of added NO_3_^−^ from Zn(NO_3_)_2_. H_2_O has the same effect as the NO_3_^−^ from the LDH metal salts. Carbonate ions will preferentially form in the interlayer region due to the higher charge density [45], therefore regardless of the presence of additional nitrate in reactor 2, no changes were observed. 

From Table 9, the specific surface area and morphology of LDHs does not appear to be affected by the residual Zn^2+^ or NO_3_^−^ (from the simulated ZnO synthesis experiments). However, the concentration of NaOH, in the second stage, does have a significant impact. Samples produced with higher concentration of NaOH (LDH6 and LDH8) show lower specific surface area than LDH_initial_ and significantly lower specific surface area than LDH5 and LDH7. Table 1 illustrates the synthesis conditions for the respective LDHs, and it appears that reducing the NaOH concentration to a concentration that matches the OH^−^/(M^II^ + M^III^) ratio of 1.5 rather than 2.5 causes a dramatic increase in specific surface area. This can be linked to changes in the morphology of the LDH samples. LDH5 exhibits a more fragmented structure consisting of smaller agglomerates with more gaps between them, whereas LDH6 has the more typical structure seen from the continuous synthesis of Mg_2_Al-CO_3_ LDHs. Agglomeration and stacking produce a block-like structure of large sheets, which cause the reduced specific surface area compared with the LDH produced at lower NaOH concentration. Specific surface area appears to be affected more by agglomeration and stacking of platelets rather than by crystal size. The reduced NaOH concentration in the feed to the second reactor causes a reduction in Mg(OH)_2_ precipitation in the LDH Mg(OH)_2_ typically precipitates at higher pH ranges than Al(OH)_3_ and Zn(OH)_2_ [41]. The concentration of Zn(OH)_2_ is negligible in the sample prepared using Zn^2+^ reaction liquor (LDH4), but it is present however in small quantities in LDH7 and LDH8. However, there is no indication from Table 9 that the Zn has a significant impact on the physicochemical properties of the LDH. Total mass loss for LDH4, LDH7 and LDH8 was 48%, 47%, and 48%, respectively, during thermal decomposition (Figure S7 in ESI). The low mass loss in LDH7 is attributed to mass loss that is 1 to 2% lower for each mass loss event compared with LDH4. The shape of the TGA profiles indicates a significant difference in thermal stability when the M^II^/M^III^ ratio is disrupted, as a result of changes in NaOH concentration. Substantial mass loss due to the breakdown of the layered structure occurs between 300 and 400 °C for samples LDH6 and LDH8, which were produced with the higher NaOH concentration. Samples produced with lower NaOH content start to break down below 300 °C. Moisture loss is also a much more rapid process for samples LDH4, LDH5, and LDH7, occurring typically <100 °C. The changes in thermal stability are due to the disruption of the sheets, caused by the changes in M^II^/M^III^ ratio, which weakens the overall structure of the LDH.

## 4. Conclusions

The results obtained from this research suggest that, for the first time, a one-step continuous synthesis method of a core-shell ZnO-LDH composite material has been achieved. However, a pure two-phase composite material was not fully synthesised, as there was an indication of the presence of Zn_2_Al-CO_3_, as well as single phase ZnO and Mg_2_Al-CO_3_ mixtures. The mass fraction of the LDH in the ZnO-LDH material was found to be approximately 0.77 from thermal decomposition studies. The relative Mg/Al ratio of the ZnO-LDH was reduced to 1.27 from the 1.97 seen in LDH_initial_. This change was due to the formation of Zn_2_Al-CO_3_ LDH from residual Zn^2+^ ions unreacted from the first reactor and the dissolution of ZnO as a result of increased temperature and reduce pH in reactor 2. The lower NaOH reduced the formation of Mg(OH)_2_ in the second reactor significantly impacted the resultant M^II^/M^III^ ratio. The CDL for the ZnO and ZnO-LDH were found to be 25 nm and 42 nm, respectively. The change in crystal size of ZnO was likely to be a result of the dissolution and re-precipitation of ZnO from smaller spherical particles into larger rod-shaped particles followed by some further precipitation of residual Zn^2+^ ions. The specific surface area of ZnO-LDH was higher than that of the single-phase powders, owing to the difference in the stacking arrangement between the single phase LDH and the LDH phase in the ZnO-LDH composite material. The open, “flower-like” pattern of LDH in the composite material allowed for a better adsorption capacity or adsorption rate compared to the single phase LDH. However, single-phase Mg_2_Al-CO_3_ LDHs synthesised at a lower NaOH concentrations (OH^−^/M^II^ + M^III^ = 1.5) resulted in a significant increase in surface (to above 250 m^2^ g^−1^) regardless of the presence of any counter ions. More work is required to optimise the synthesis process (to ensure near-perfect coverage of the ZnO core with LDH platelets). In addition, their capability for adsorption and photodegradation of organic dyes should be explored.

## Figures and Tables

**Table 1:** Synthesis conditions for layered double hydroxides (LDHs) and composite.

Sample	Reactor	Material	[NaOH] (mol L^−1^)	Down-Flow (mL min^−1^)	[M^II^ + M^III^] (mol L^−1^)	Up-Flow (mL min^−1^)
ZnO	1	ZnO	0.050	20	0.05	10
LDH_initial_	1	Mg_2_Al-CO_3_	0.125	20	0.10	10
^†^ LDH_mix_	-	mixture of ZnO and LDH_initial_	-	-	-	-
ZnO-LDH	1 - 2	ZnO - Mg_2_Al-CO_3_	0.05 - 0.15	40	0.05 - 0.05	20
LDH-only experiments						
LDH3	2	Mg_2_Al-CO_3_	0.25	40	0.05	20
* LDH4	2	Mg_2_Al-CO_3_	0.15	40	0.05	20
LDH5	2	Mg_2_Al-CO_3_	0.15	40	0.05	20
LDH6	2	Mg_2_Al-CO_3_	0.25	40	0.05	20
LDH7	2	Mg_2_Al-CO_3_	0.15	40	0.05	20
LDH8	2	Mg_2_Al-CO_3_	0.25	40	0.05	20

* Recovered reaction liquor from ZnO synthesis used to flow through reactor 1. ^†^ Dry ZnO/LDH_initial_ powders mixed at 23%:77% ratio of ZnO:LDH.

**Table 2:** Lattice parameters of pure and composite materials.

Sample	Miller Indices	^a^ Lattice Parameter a (nm)	Miller Indices	^a^ Lattice Parameter c (nm)
ZnO	010	0.33	002	0.52
LDH_initial_	110	0.30	003	2.32
ZnO-LDH	010 110	0.32 ^a^ 0.31 ^b^	002 003	0.52 ^a^ 2.28 ^b^

^a^ Lattice parameters for ZnO phase. ^b^ Lattice parameters for Mg_2_Al-CO_3_ phase.

**Table 3:** Crystal domain length and particle size of ZnO, Mg_2_Al-CO_3_ (LDH_initial_), and ZnO-LDH composite.

Sample	XRD			TEM	
	CDL (012) (nm)	CDL (003) (nm)	CDL (110) (nm)	^b^ Particle Size (nm)	^b^ Particle Size (nm)
ZnO	25	-	-	22 ± 14	-
LDH_initial_	-	8	25	-	34 ± 34
ZnO-LDH	42	12	27	53 ± 40	51 ± 22

^b^ Average particle size from TEM micrographs with standard deviation of from Gaussian distribution (*n* = 3).

**Table 4:** Specific surface area, pore diameter, and cumulative pore volume of ZnO, Mg2Al-CO3 (LDHinitial), ZnO-LDH composite, and a mix of ZnO and LDHinitial (LDHmix).

Sample	S_BET_ (m^2^ g^−1^)	Pore Diameter (nm)	Pore Volume (cm^−3^ g^−1^)
ZnO	53.0 ± 0.1	17.6	0.3
LDH_initial_	58.2 ± 0.3	4.0	0.1
LDH_mix_	39.6 ± 0.1	7.2	0.1
ZnO-LDH	76.2 ± 0.1	17.0	0.7

**Table 5:** Band gap energy of the components and hybrid structure.

Sample	Band Gap (eV)
ZnO	3.21
LDH_initial_	5.24
LDH_mix_	3.22
ZnO-LDH	3.31

**Table 6:** Changes to crystal domain length (CDL) and Reynolds number in reactors 1 and 2.

Sample	Reactor	Flow Rate (mL min^−1^)	Re	CDL (003) (nm)	CDL (110) (nm)
LDH_initial_	1	30	235	8	25
LDH3	2	60	297	7	30

**Table 7:** Specific surface area (S_BET_) and pore characteristics of Mg_2_Al-CO_3_ LDH synthesised from reactors 1 and 2.

Sample	S_BET_ (m^2^ g^−1^)	BJH (Barrett, Joyner, and Halenda) Pore Diameter (nm)	BJH Pore Volume (cm^3^ g^−1^)
LDH_initial_	58.2 ± 0.3	3.5	0.07
LDH3	20.1 ± 0.1	4.2	0.04

**Table 8:** Scherrer approximation of crystal domain length (CDL).

Sample	CDL (003)/nm	CDL (110)/nm
LDH4	6	25
LDH5	6	26
LDH6	7	25
LDH7	5	27
LDH8	8	27

**Table 9:** Specific surface area (SBET) and pore size for samples isolating residual ions from ZnO synthesis.

Sample	S_BET_ (m^2^ g^−1^)	Pore Diameter (nm)	Pore Volume (cm^3^ g^−1^)
LDH4	266.8 ± 0.4	16.85	1.36
LDH5	270.2 ± 0.5	15.87	1.29
LDH6	22.6 ± 0.1	3.89	0.06
LDH7	271.7 ± 0.5	14.55	1.20
LDH8	26.1 ± 0.2	4.42	0.05

## Supplementary Materials

The following are available online at https://www.mdpi.com/2079-4991/10/10/2052/s1, Figure S1: Overview of all experiments, Figure S2: Scanning electron images (at a magnification of x100) of (a) LDHinitial, (b) ZnO-LDH and EDAX spectra, (c) LDHinitial, (d) ZnO-LDH, Table S1: EDX spectra results outlining atomic and mass fractions of elements in ZnO-LDH composite, Figure S3: (a) UV-Vis DRS, b) Kubelka–Munk plot, Figure S4: XRD patterns for MgAlCO_3_ made in reactor 1 and reactor 2, Figure S5: SEM micrographs depicting differences in microstructure and morphology between samples LDHinitial (a, b) and LDH3 (c, d), Figure S6: Diffraction patterns for LDH samples LDH4-LDH8, Figure S7: Derivative mass loss profiles of LDHs produced with varying precursor ions and OH- concentration, Table S2: Weight fraction of metals in each LDH structure and residual metal content.
