# Supplementary material for: Towards the Continuous Hydrothermal Synthesis of ZnO@Mg2Al-CO3 Core-Shell Composite Nanomaterials
Source: Nanomaterials (Basel). 2020 Oct 16;10(10):2052. doi: 10.3390/nano10102052 (PMC7602976; doi:10.3390/nano10102052)
Supplement: Supplementary file 1 [file nanomaterials-10-02052-s001.pdf]

Supplementary Information

Towards the Continuous Hydrothermal Synthesis of $ZnO@Mg_2Al-CO_3$ Core-Shell Composite Nanomaterials

Ian Clark [1,2], Jacob Smith [1,2], Rachel L. Gomes [2] and Edward Lester [1,*]

1 Advanced Materials Research Group, Faculty of Engineering, University of Nottingham, University Park, Nottingham, NG7 2RD, United Kingdom; ian.clark@nottingham.ac.uk (I.C.); jacob.smith@nottingham.ac.uk (J.S.)

2 Food, Water, Waste Research Group, Faculty of Engineering, University of Nottingham, University Park, Nottingham, NG7 2RD, United Kingdom; Rachel.gomes@nottingham.ac.uk

* Correspondence: edward.lester@nottingham.ac.uk

Figure S1 – overview of all experiments

ZnO

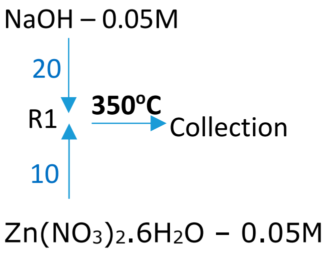

ZnO – Simple ZnO synthesis in reactor 1

LDH$_{initial}$

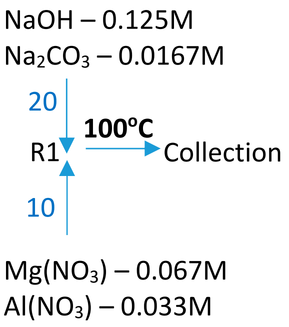

LDH1 – Simple Mg_2Al-CO_3 synthesis in reactor 1

LDH_{mix}

An 'artificial' mix of $LDH_{initial}$ and ZnO from the separate experiments described above

ZnO-LDH

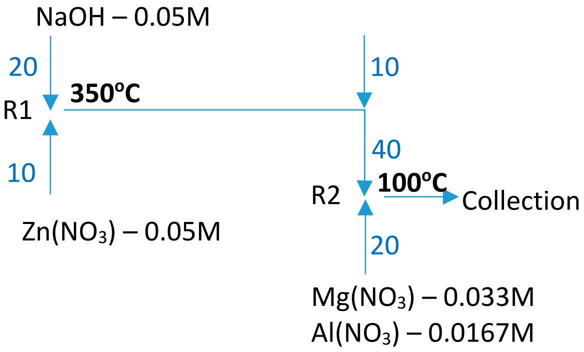

ZnO-LDH the hybrid synthesis whereby ZnO is made in reactor 1 which then flows into reactor 2 where the Mg_2Al-CO_3 is synthesised

LDH3

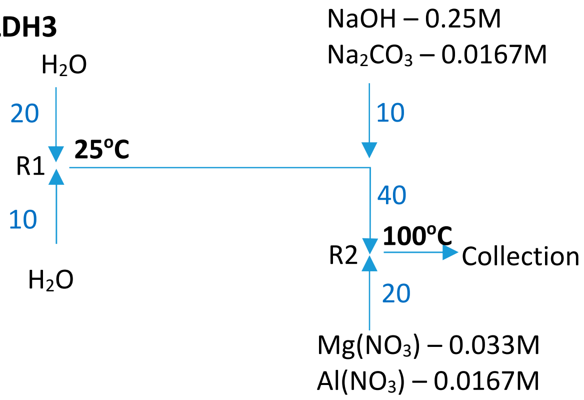

LDH3 –Mg_2Al-CO_3 synthesis in reactor 2 with scaled concentration to match flow rate changes

LDH4

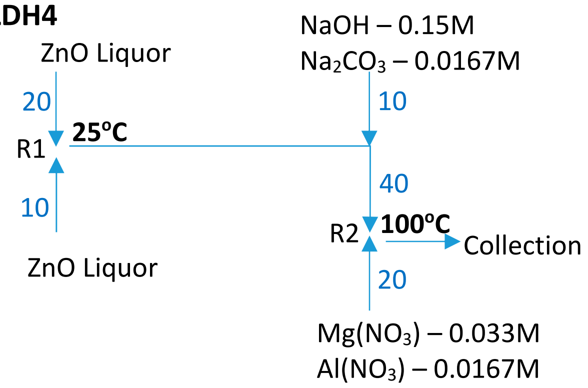

LDH4 –Mg_2Al-CO_3 synthesis in reactor 2 with scaled concentration to match concentration of LDH post ZnO synthesis. ZnO liquor to evaluated effect of residual Zn^{2+} and any potential OH^- from R1

LDH5

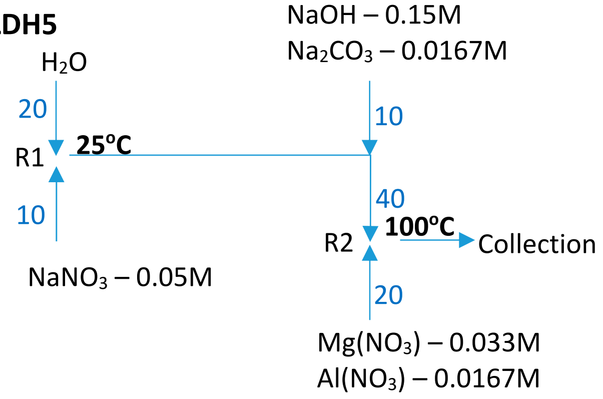

LDH5 –Mg_2Al-CO_3 synthesis in reactor 2 with scaled concentration to match concentration of LDH post ZnO synthesis. $NaNO_3$ added to evaluate effect of NO_3^- from ZnO synthesis without the presence of Zn^{2+} or OH^- from R1

LDH6

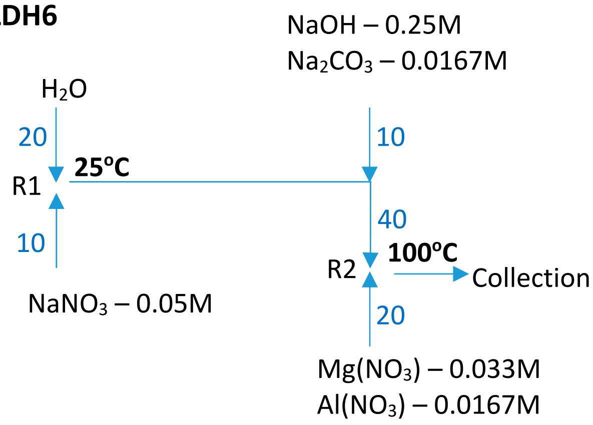

LDH6 –Mg_2Al-CO_3 synthesis in reactor 2 with scaled concentration to match concentration of LDH without ZnO synthesis – R2 conditions mirror LDH3. $NaNO_3$ added to evaluate effect of NO_3^- from ZnO synthesis without the presence of Zn^{2+} or OH^- from R1

LDH7

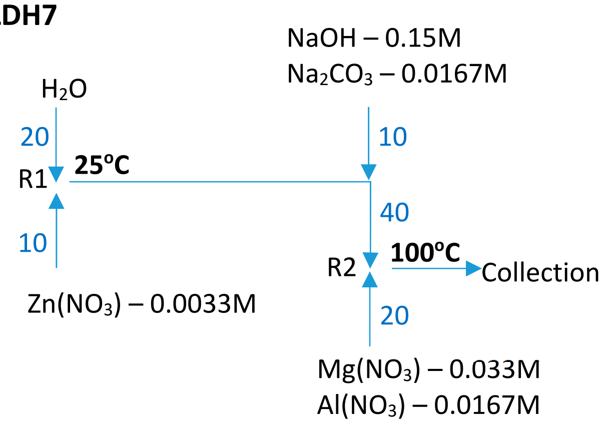

LDH7 –Mg_2Al-CO_3 synthesis in reactor 2 with scaled concentration to match concentration of LDH with ZnO synthesis. $Zn(NO_3)$ added to evaluate effect of residual Zn^{2+} from ZnO synthesis without the presence of OH^- from R1

LDH8

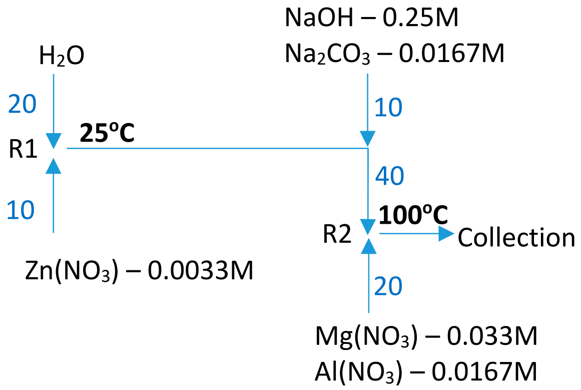

LDH8 –Mg_2Al-CO_3 synthesis in reactor 2 with scaled concentration to match concentration of LDH without ZnO synthesis – R2 conditions mirror LDH3. $Zn(NO_3)$ added to evaluate effect of Zn^{2+} from ZnO synthesis without the presence of OH^- from R1

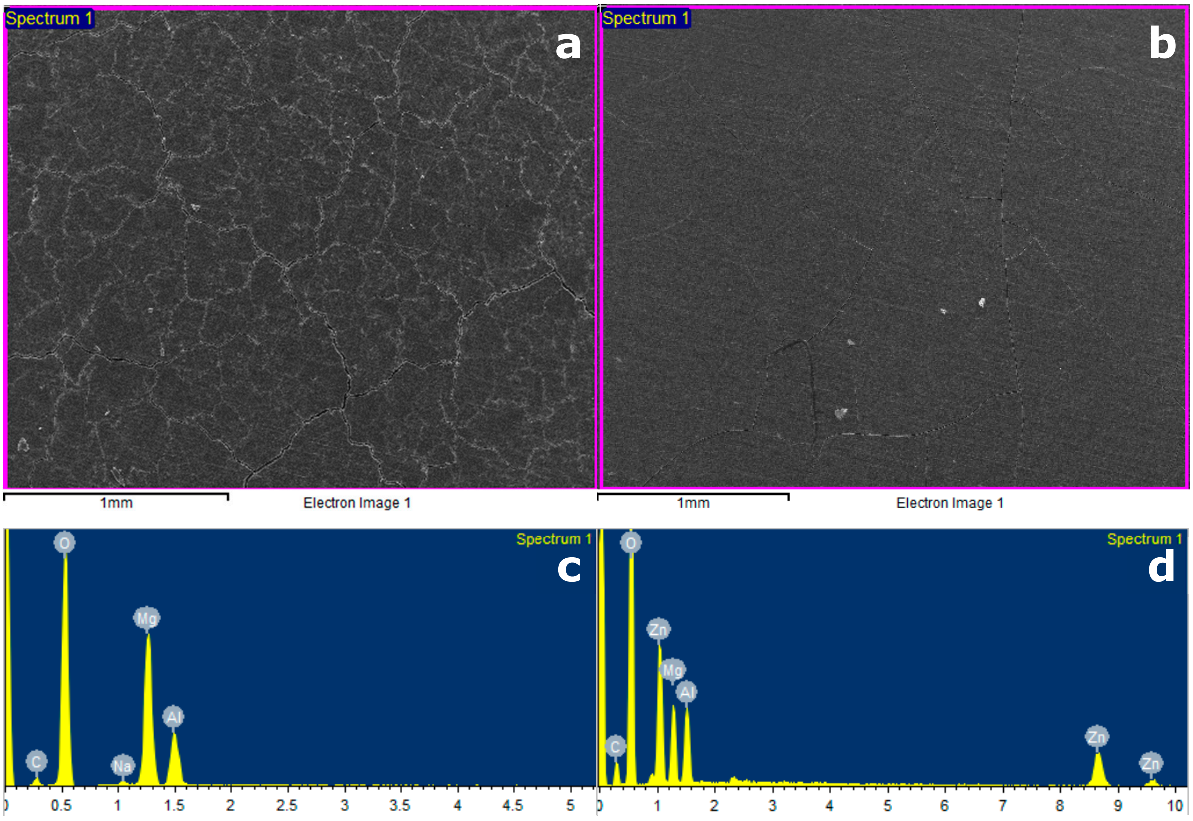

Figure S2 Scanning electron images (at a magnification of x100) of a) $LDH_{initial}$, b) ZnO-LDH and EDAX spectra c) $LDH_{initial}$, d) ZnO-LDH

Table S1 EDX spectra results outlining atomic and mass fractions of elements in ZnO-LDH composite

Element	Atomic Fraction	Weight Fraction	Compound Fraction	Formula
Mg	17.68	16.19	26.42	MgO
Al	13.55	13.77	27.25	Al_2O_3
Zn	15.39	37.88	46.34	ZnO
O	53.39	32.17		

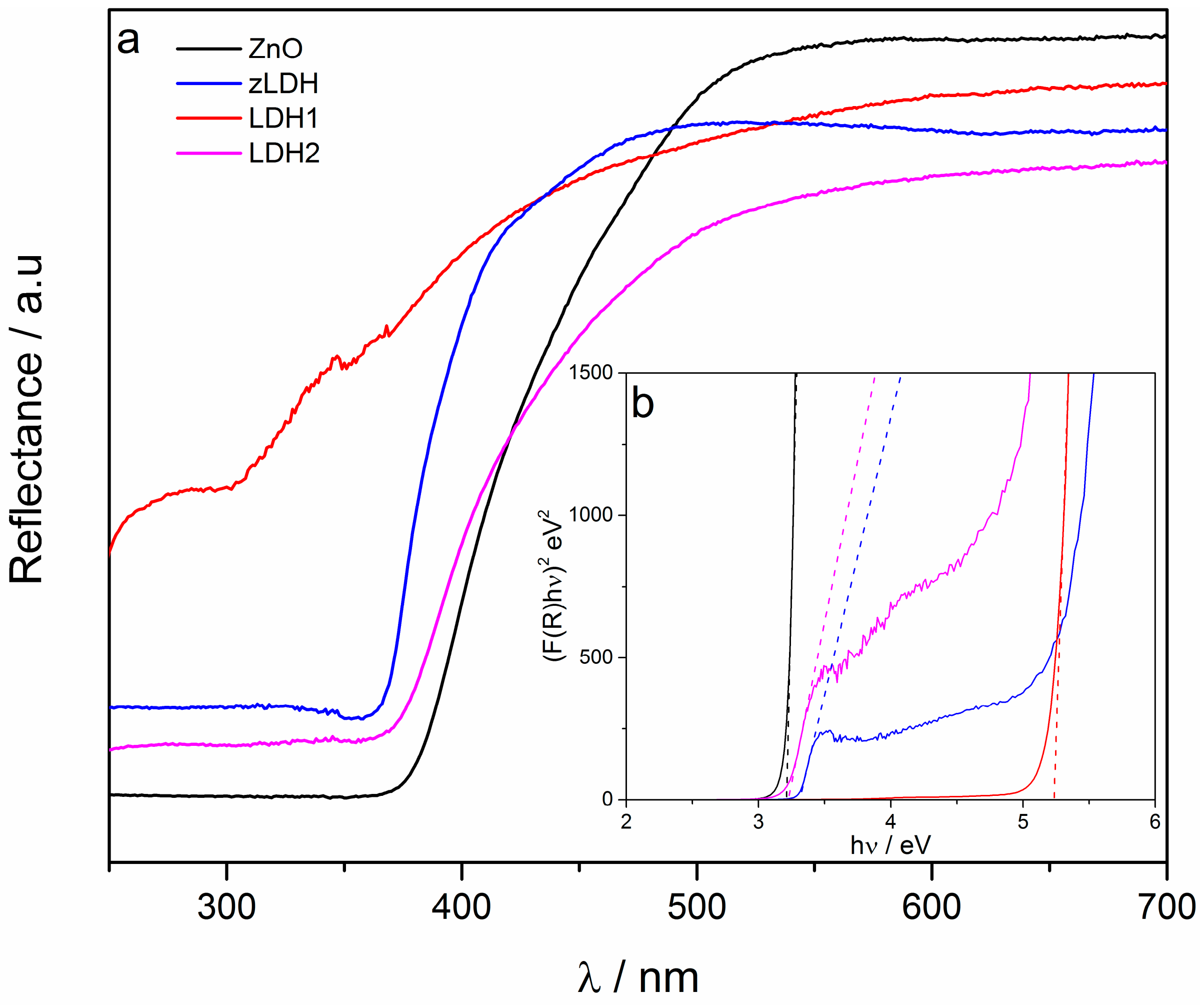

Figure S3 a) UV-Vis DRS, b) Kubelka-Munk plot

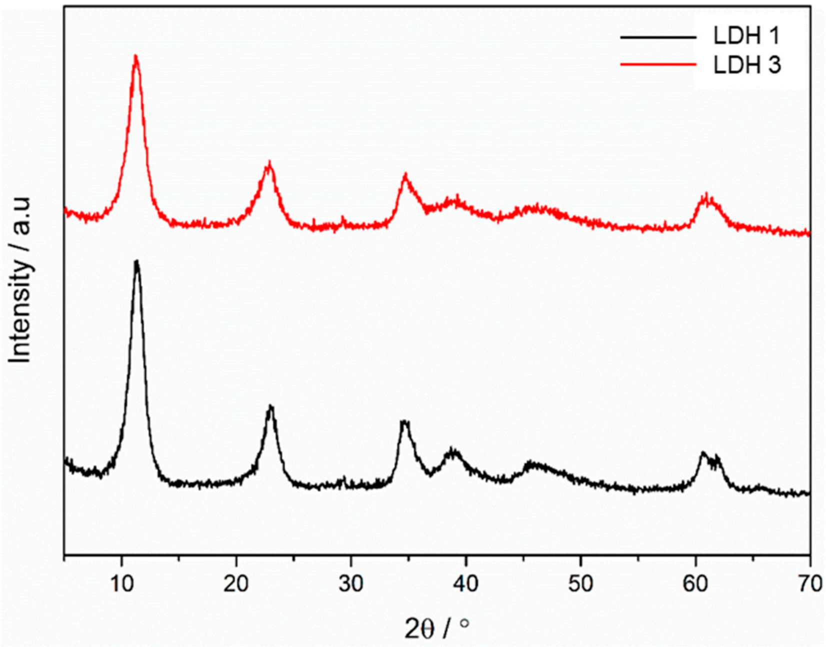

Figure S4 – XRD patterns for $MgAlCO_3$ made in reactor 1 (LDH 1) and reactor 2 (LDH 3)

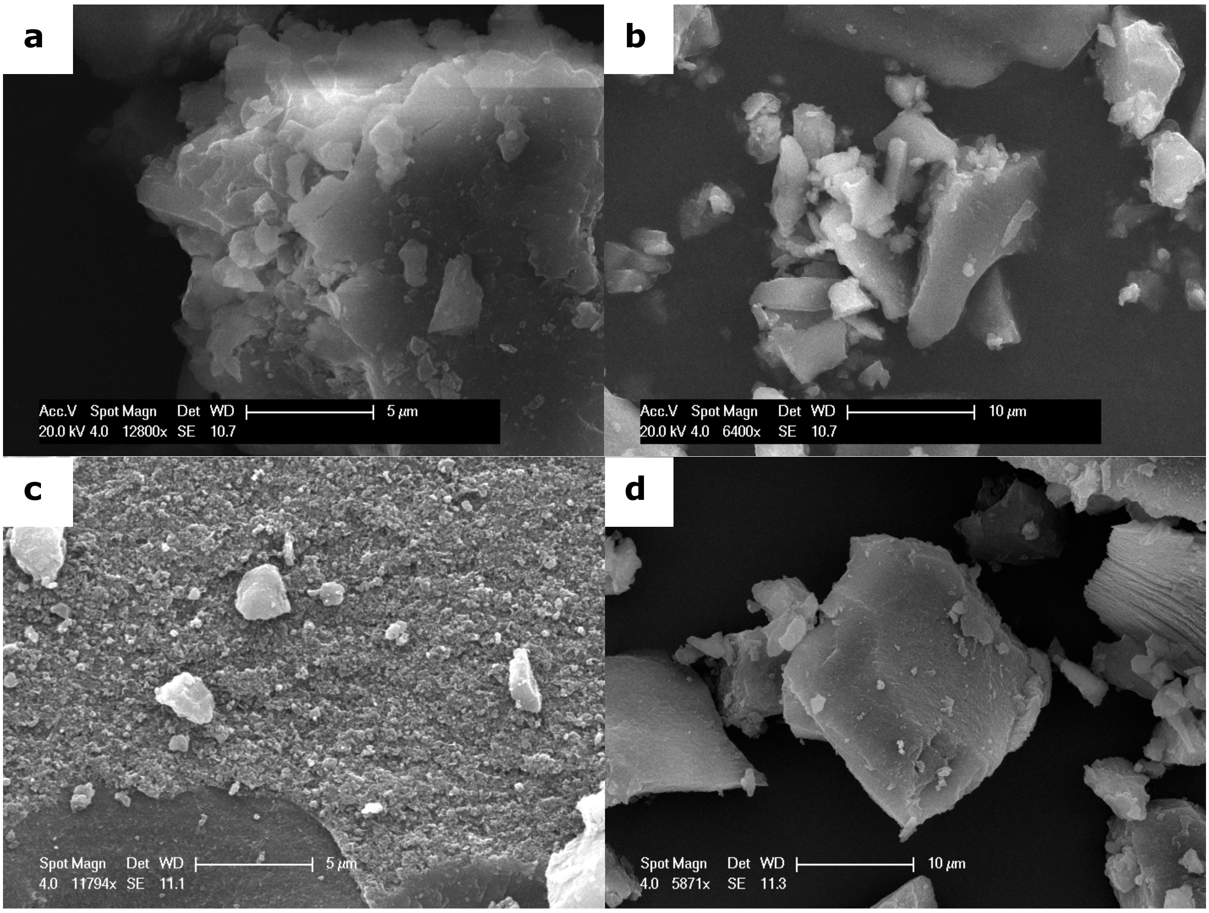

Figure S5 SEM micrographs depicting differences in microstructure and morphology between Samples $LDH_{initial}$ (a, b) and LDH3 (c, d)

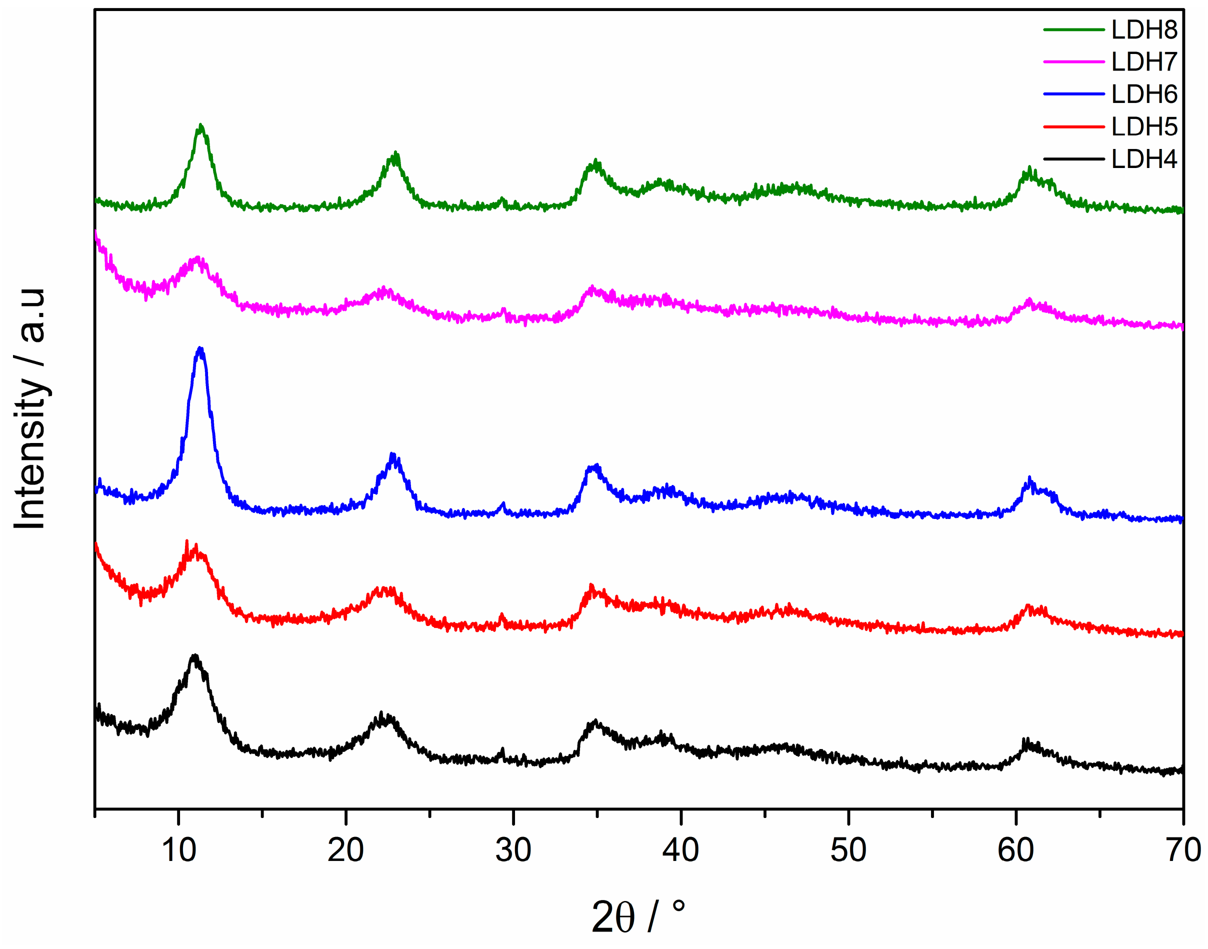

Figure S6 Diffraction patterns for LDH samples LDH4-LDH8

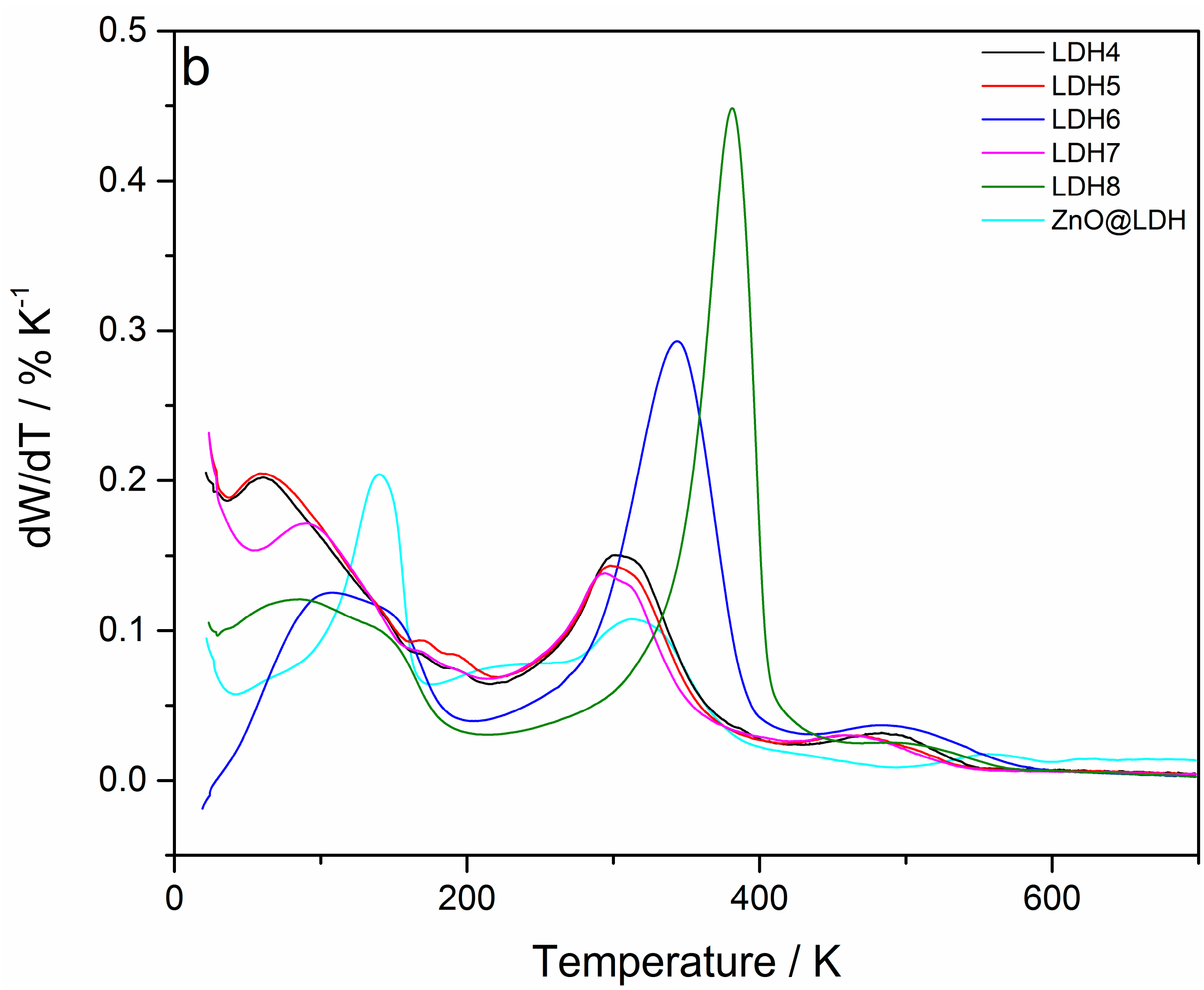

Figure S7 Derivate mass loss profiles of LDHs produced with varying precursor ions and OH-concentration

Table S2 Weight fraction of metals in each LDH structure and residual metal content

Sample	LDH Content			
	Mg %	Al %	Zn %	Mg/Al
$LDH_{initial}$	30.30	15.40		1.97
LDH3	30.19	15.85		1.90
LDH4	23.42	21.26		1.10
LDH5	21.22	23.02		0.92
LDH6	30.88	15.3		2.02
LDH7	20.25	22.72	1.35	0.89
LDH8	30.68	14.42	1.3	2.13
